# Natural Proline-Rich Cyclopolypeptides from Marine Organisms: Chemistry, Synthetic Methodologies and Biological Status

**DOI:** 10.3390/md14110194

**Published:** 2016-10-26

**Authors:** Wan-Yin Fang, Rajiv Dahiya, Hua-Li Qin, Rita Mourya, Sandeep Maharaj

**Affiliations:** 1 School of Chemistry, Chemical Engineering and Life Science, Wuhan University of Technology, Wuhan 430070, China; wanyinfang@whut.edu.cn; 2 Laboratory of Peptide Research and Development, School of Pharmacy, Faculty of Medical Sciences, The University of the West Indies, Saint Augustine, Trinidad and Tobago, West Indies; Sandeep.Maharaj@sta.uwi.edu; 3 School of Pharmacy, College of Medicine and Health Sciences, University of Gondar, Gondar 196, Ethiopia; ritz_pharma@yahoo.co.in

**Keywords:** proline-rich cyclic peptide, marine sponge, marine tunicate, peptide synthesis, stereochemistry, lipophilicity parameter, pharmacological activity

## Abstract

Peptides have gained increased interest as therapeutics during recent years. More than 60 peptide drugs have reached the market for the benefit of patients and several hundreds of novel therapeutic peptides are in preclinical and clinical development. The key contributor to this success is the potent and specific, yet safe, mode of action of peptides. Among the wide range of biologically-active peptides, naturally-occurring marine-derived cyclopolypeptides exhibit a broad range of unusual and potent pharmacological activities. Because of their size and complexity, proline-rich cyclic peptides (PRCPs) occupy a crucial chemical space in drug discovery that may provide useful scaffolds for modulating more challenging biological targets, such as protein-protein interactions and allosteric binding sites. Diverse pharmacological activities of natural cyclic peptides from marine sponges, tunicates and cyanobacteria have encouraged efforts to develop cyclic peptides with well-known synthetic methods, including solid-phase and solution-phase techniques of peptide synthesis. The present review highlights the natural resources, unique structural features and the most relevant biological properties of proline-rich peptides of marine-origin, focusing on the potential therapeutic role that the PRCPs may play as a promising source of new peptide-based novel drugs.

## 1. Introduction

An interesting class of marine cyclic peptides is represented by the proline-rich compounds usually containing more than six or seven amino acid residues. The role of proline in these molecules has been linked to the control of the conformation of the molecule in solution because of the restricted φ of proline. The proline-rich cyclic peptides (PRCPs) are formed by linking one end of the peptide and the other with an amide bond or other chemically-stable bonds. Some of them are used in the clinic, e.g., gramicidin S and tyrocidine with bactericidal activity, while others are in clinical trials, e.g., dehydrodidemnin B, and most of them originate from natural resources. Although the literature is enriched with reports concerned with marine-derived linear proline-rich bioactive peptides [1,2,3,4,5], e.g., dolastatin 15, kurahyne B, jahanyne, cemadotin, koshikamide A_1_, etc., PRCPs from marine resources are becoming popular and attracting the attention of scientists nowadays, due to their unique structural features and a wide range of the biological properties, like cytotoxicity [6], antibacterial activity [7], antifungal activity [8], immunosuppressive activity [9], anti-inflammatory activity [10], anti-HIV activity [11], repellent (antifouling) activity [12], antitubercular activity [13] and antiviral activity [14], associated with them. PRCPs include a large and heterogeneous group of small to large-sized oligopeptides characterized by the presence of proline units often constituting peculiar sequences, which confers them a typical structure that determines the various biological functions endowed by these molecules. As several features make PRCPs attractive lead compounds for drug development, as well as nice tools for biochemical research, scientists are focusing and giving diverse efforts to develop biologically-active proline-rich cyclic peptide compounds.

### 1.1. Natural Resources

**Figure 1:**
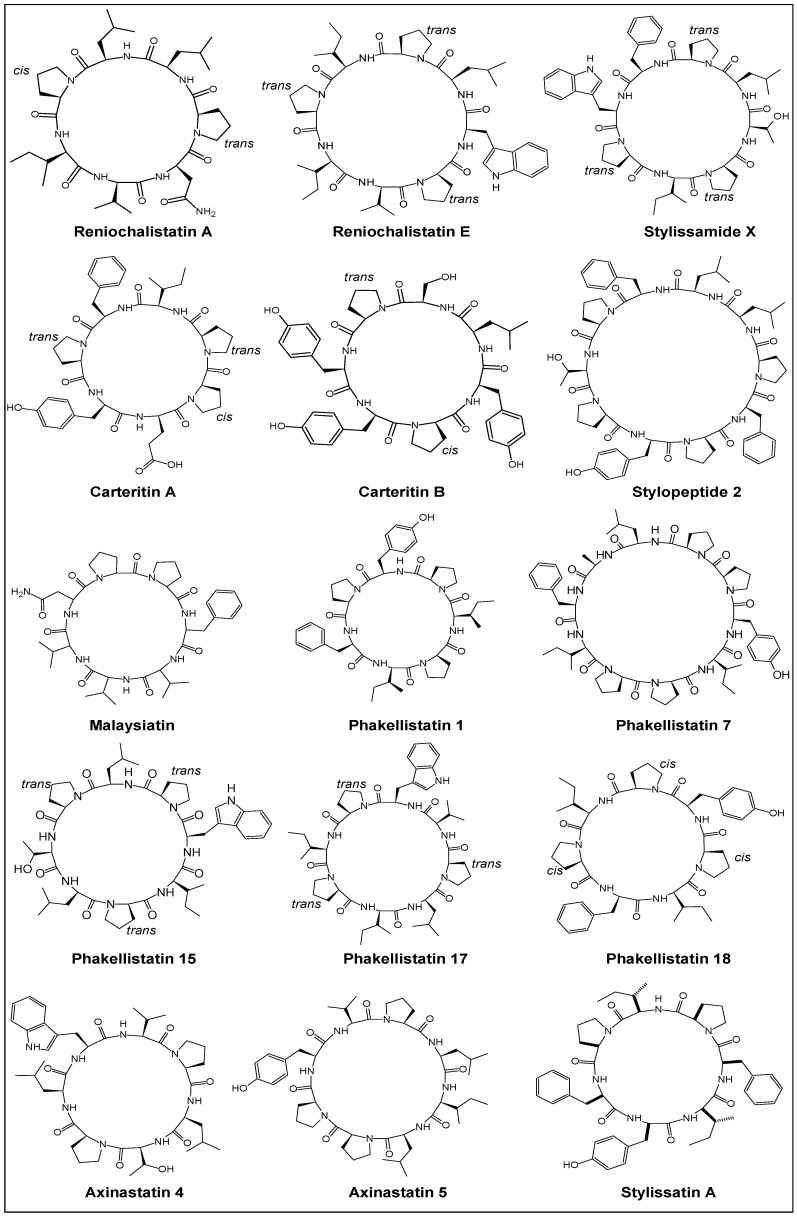

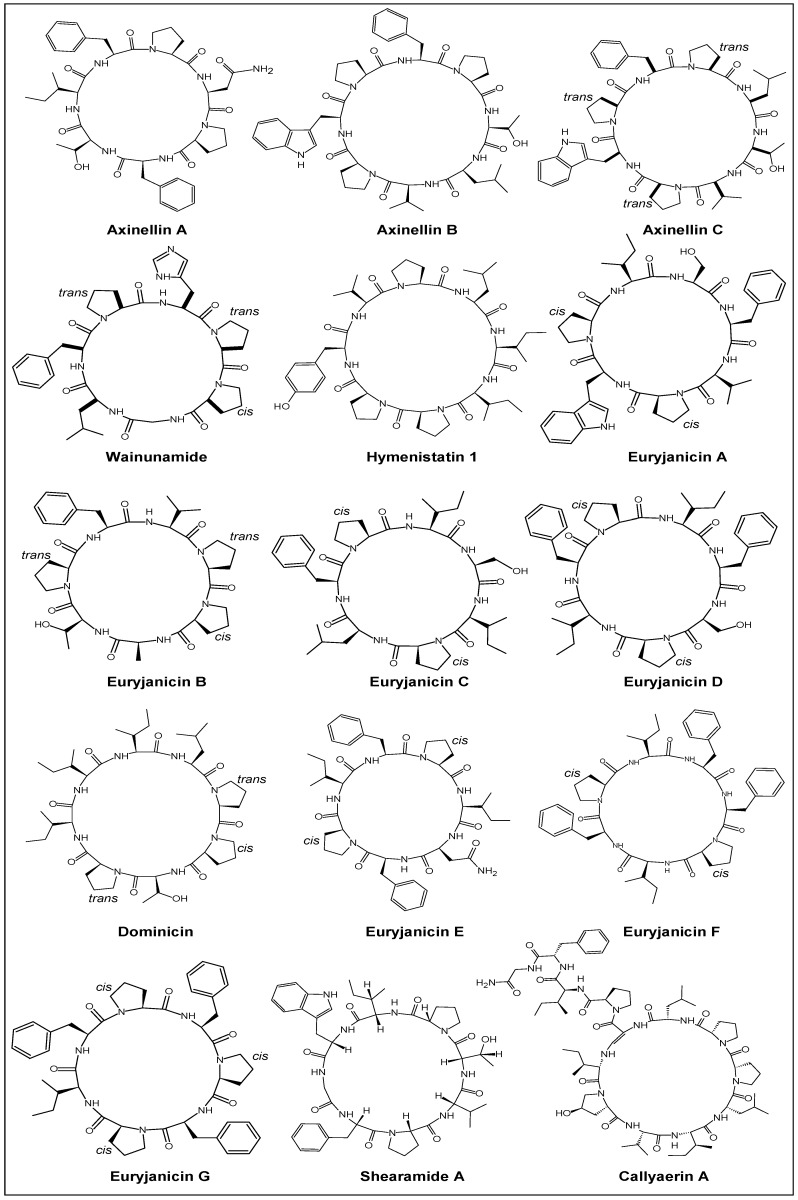
Proline-rich cyclic peptides (PRCPs) from marine sponges.

Various natural sources of PRCPs include marine sponges, ascidians, different genera of cyanobacteria and higher plants. One of the potent resources is sessile aquatic animals, i.e., sponges like Kenyan sponge *Callyspongia abnormis* [15], Dominican sponge *Eurypon laughlini* [16], Indonesian sponge *Callyspongia aerizusa* [17], sponge *Ircinia* sp. [18], Jamaican sponge *Stylissa caribica* [19], Yongxing Island sponge *Reniochalina stalagmitis* [20], Vanuatu sponge *Axinella carteri* [21], Korean sponge *Clathria gombawuiensis* [22], Fijian sponge *Stylotella aurantium* [23], Papua New Guinea sponge *Stylissa massa* [24], South China sponge *Phakella fusca* [25], Lithistid sponge *Scleritoderma nodosum* [26], Borneo sponge *Pseudaxinyssa* sp. [27], Philippines sponge *Myriastra clavosa* [28], Papua New Guinea sponge *Stylotella* sp. [29], Comoros sponge *Axinella* cf. *carteri* [30], Okinawan sponge *Hymeniacidon* sp. [31], Indo-Pacific sponges *Phakellia costata* and *Stylotella aurantium* [32], Indonesian sponge *Stylissa* sp. [33], Red sea sponge *Stylissa carteri* [34], Western Pacific Ocean sponge *Hymeniacidon* sp. [35], Puerto Rican sponge *Prosuberites laughlini* [36], Micronesian sponge *Cribrochalina olemda* [37], Indonesian sponge *Sidonops microspinosa* [38], Palau sponge *Axinella* sp. [39], etc. The structures of various proline-rich cyclopolypeptides from marine sponges are compiled in Figure 1.

**Figure 2:**
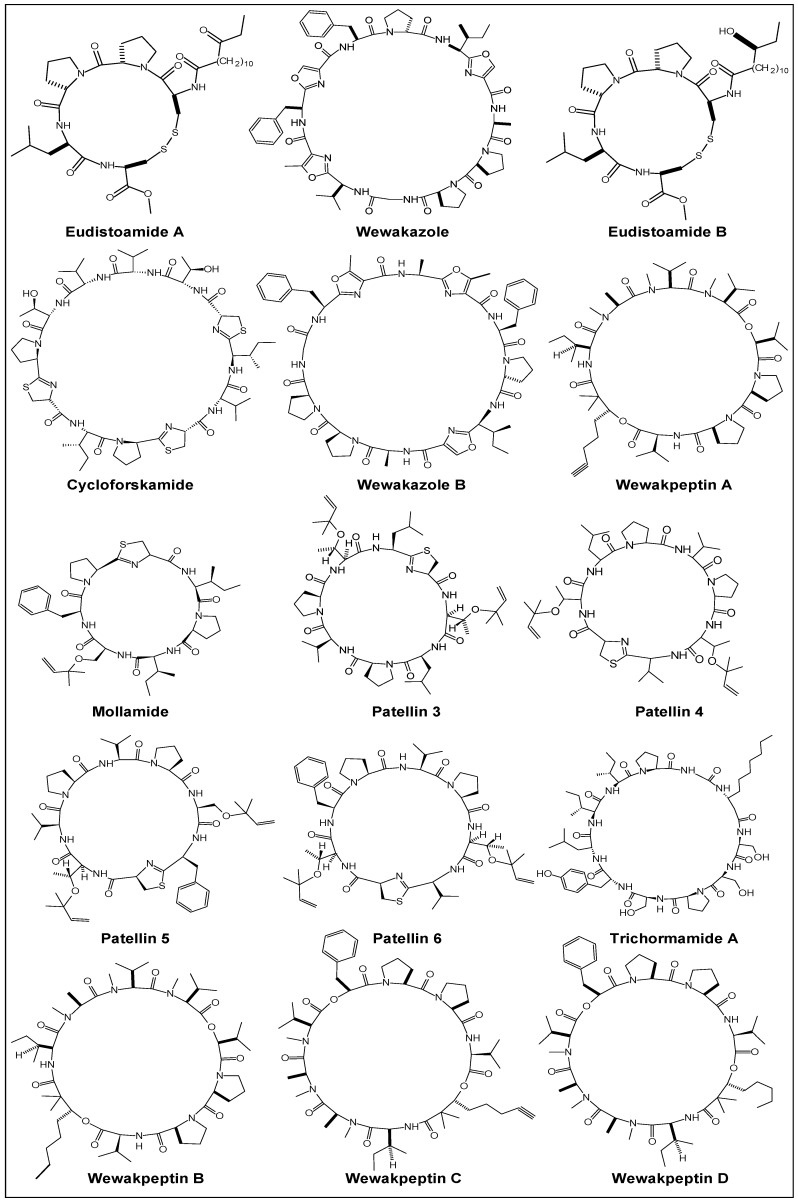
PRCPs from marine ascidians (tunicates) and cyanobacteria.

Other sources of proline-rich cyclooligopeptides are marine tunicates, like compound ascidian *Didemnum molle* [40], Ishigaki Island sea slug *Pleurobranchus forskalii* [41], Fijian ascidian *Eudistoma* sp. [42], Caribbean tunicate *Trididemnum solidum* [43], unidentified Brazilian ascidian (family Didemnidae) [44], Mediterranean ascidian *Aplidium albicans* [45], cyanobacteria like Papua New Guinea cyanobacterium *Lyngbya semiplena* [46], Red Sea cyanobacterium *Moorea producens* [47], Florida Everglades cyanobacterium *Lyngbya* sp. [48], Northern Wisconsin cyanobacterium *Trichormus* sp. UIC 10339 [49], toxic cyanobacterium *Nostoc* sp. 152 [50], Kenyan cyanobacterium *Lyngbya majuscule* [51], mollusks like Papua New Guinea mollusk (sea hare) *Dolabella auricularia* [52] and alga like Indonesian red alga (Rhodophyta) *Ceratodictyon spongiosum* containing the symbiotic sponge *Sigmadocia symbiotica* [10]. Structures of diverse proline-rich cyclopeptides from marine tunicates and cyanobacteria are tabulated in Figure 2. Besides this, proline-containing cyclooligopeptides are also obtained from roots, stems, barks, seeds, fruit peels of higher plants, as well as from bacteria and fungi [53,54,55,56,57,58,59,60,61,62,63,64,65,66].

Purification procedures of PRCPs isolated from sea animals, like ascidians, sponges and mollusk, usually include initial extraction with methanol (MeOH), partitions of these extracts with organic solvents of increasing polarities to render diverse organic fractions and chromatographic steps on silica and Sephadex LH-20 columns, as well as the use of reversed phase C18 HPLC for the final purification [67].

### 1.2. Stability and Comparison with Linear Peptides

Linear peptides that contain less than 10 amino acid residues are especially flexible in solution. Once the length of linear peptides extends to between 10 and 20 amino acid residues, random linear peptide sequences can begin to obtain secondary structures, including α-helices, turns and β-strands. These secondary structures impose constraints that reduce the free energy of linear peptides and limit their conformations to those that may be more biologically active. The constraints imposed by cyclization force cyclic peptides to adopt a limited number of molecular conformations in solution. Generally, if cyclization limits conformations to those required for optimum receptor binding, these cyclic peptides would be more useful compared with their linear counterparts that can adopt more conformations, which are not useful for receptor binding. Cyclization has been shown to increase the propensity for β-turn formation in peptides, which is of vital utility since β-turns are often found in native proteins. Although peptide cyclization generally induces structural constraints, the site of cyclization within the sequence can affect the binding affinity of cyclic peptides.

In the case of proline, which is a proteinogenic amino acid with a secondary amine that does not follow along with the typical Ramachandran plot, the ψ and φ angles about the peptide bond have fewer allowable degrees of rotation due to the ring formation connected to the beta carbon. As a result, it is often found in “turns” of peptides/proteins, as its free entropy (ΔS) is not as comparatively large as other amino acids, and thus, in a folded form vs. unfolded form, the change in entropy is less. Furthermore, proline is rarely found in α and β structures, as it would reduce the stability of such structures, because its side chain α-N can only form one hydrogen bond.

Further, the hydroxylation of proline by prolyl hydroxylase and other additions of electron-withdrawing substituents, such as fluorine, increases the conformational stability of collagen significantly. Hence, the hydroxylation of proline is a critical biochemical process for maintaining the connective tissue of higher organisms. Polypeptide chains containing proline lack the flexibility of other peptides, because the proline ring has only one available angle for backbone rotation. Rotation occurs around the angles φ, ψ and ω [68,69].

The cyclization of linear peptide sequences can create constrained geometries that can alter the specificity of cyclic peptides to different isoforms or subtypes of targeted receptors. Peptides can be cyclized in order to reduce the overall numbers of interchanging conformers in the hope of limiting them to those selective for the desired receptors while avoiding degradation by not forming conformers susceptible to interacting with proteolytic enzymes [70].

In general, cyclization often increases the stability of peptides [71,72], which can prolong their biological activity. This prolonged activity may even be the result of additional resistance to enzymatic degradation by exoproteases that preferentially cleave near the *N*- or *C*-termini of peptide sequences. In particular, cyclization can create peptides with the ability to penetrate tumors in order to enhance the potency of anticancer drugs [73]. Cyclic peptides can potentially obtain desirable constrained geometries that are responsible for increasing their binding affinity, specificity or stability compared with their linear counterparts. Cyclic peptides are of considerable interest as potential protein ligands and might be more cell permeable than their linear counterparts due to their reduced conformational flexibility. However, it is important to note that cyclization does not necessarily lead to improvements in all of these properties, e.g., linear peptides can contain sequences that can support rigid structures without the need for cyclization [74].

## 2. Chemistry

### 2.1. Structural Features

The distinctive cyclic structure of proline’s side chain gives proline an exceptional conformational rigidity compared to other amino acids, which affects the rate of peptide bond formation between proline and other amino acids. The exceptional conformational rigidity of proline affects the secondary structure of proteins near a proline residue and may account for proline’s higher prevalence in the proteins of thermophilic organisms. Proline acts as a structural disruptor in the middle of regular secondary structure elements, such as alpha helices and beta sheets; however, proline is commonly found as the first residue of an alpha helix and also in the edge strands of beta sheets. Multiple prolines and hydroxyprolines in a row can create a polyproline helix, the predominant secondary structure in collagen [75].

**Figure 3:**
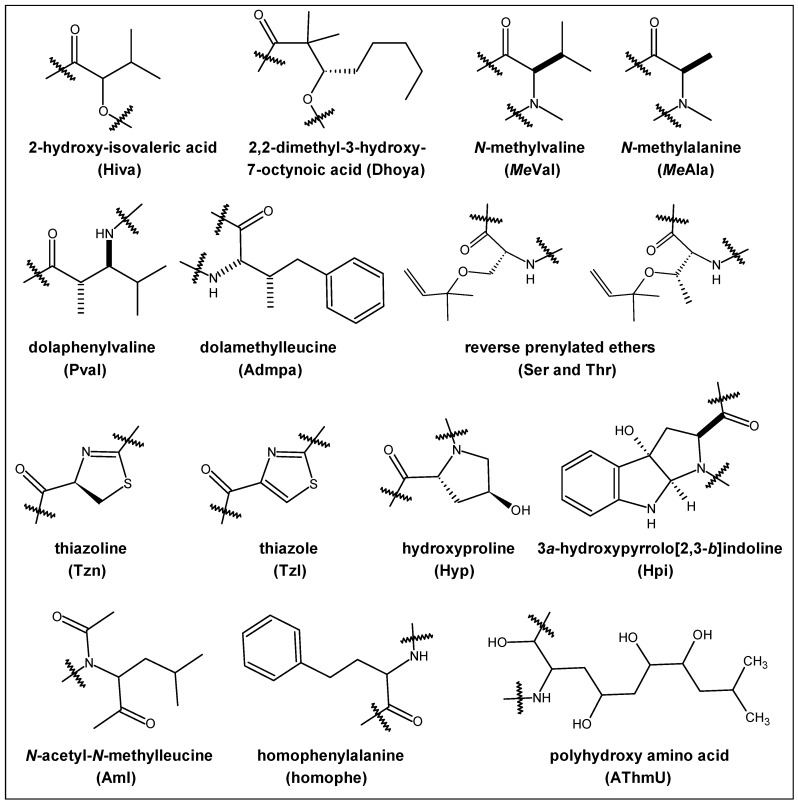
Modified amino acid moieties/heterocyclic rings present in marine-derived PRCPs.

The number of proline units in a cyclic peptide structure varies from one to five (Table 1). In addition to normal hydrophobic amino acids, marine organism-derived cyclopolypeptides rich in proline units contain modified and unusual amino acid moieties and other rings, like hydroxyproline (Hyp), (*Z*)-2,3-diaminoacrylic acid (DAA), thiazoline (Tzn), thiazole (Tzl), oxazole, methyloxazoline, reverse prenylated ethers, i.e., serine and threonine carrying a dimethylallyl ether group, *para*-hydroxystyrylamide (*p*HSA), pyroglutamic acid (pyroGlu), 3*a*-hydroxypyrrolo[2,3-*b*]indoline (Hpi), the 12-hydroxy-tetradecanoyl moiety, 2-(1-amino-2-*p*-hydroxyphenylethane)-4-(4-carboxy-2,4-dimethyl-2*Z*,4*E*-propadiene)-thiazole (ACT), *O*-methyl-*N*-sulfo-d-serine, keto-*allo*-isoleucine, methyloxazoline, β-methoxyaspartic acid, β-aminodecanoic acid, 2,2-dimethyl-3-hydroxy-7-octynoic acid (Dhoya), β-amino acid 3-amino-2-methylbutanoic acid (Maba) and 2-Hydroxy-isovaleric acid (Hiva), *O*-prenyltyrosine (Ptyr) (2*S*,3*R*,5*R*)-3-amino-2,5-dihydroxy-8-phenyloctanoic acid (Ahoa), dolaphenvaline (Pval) and dolamethylleucine (Admpa), *N*-acetyl-*N*-methylleucine (Aml), *E*- and *Z*-dehydrobutyrines (Dhb), a homophenylalanine (homophe), (2*S*,3*R*)-β-hydroxy-*p*-bromophenylalanine and *N*,*O*-dimethyl tyrosine, hydroxyisovaleric acid (Hiv) (Figure 3).

Callynormine A represents a new class of heterodetic cyclic peptides possessing an α-amido-β-aminoacrylamide cyclization functionality. Hyp forms part of the composition of cyclic endiamino peptides like callynormine A [15] and callyaerin A–D. The unusual non-proteinogenic (*Z*)-DAA moiety is characteristic of the callyaerin series of peptides callyaerins A–M, which links the cyclic peptide part of the callyaerins with a linear peptide side chain [13]. Indo-Pacific ascidian *Didemnum molle* is found to be rich in thiazole-, oxazole- and thiazoline-containing peptides, like mollamide, which share the peculiar reverse prenylated ethers of serine and threonine amino acids [40].

Furthermore, unusual amino acid residues like *p*HSA and pyroGlu were found to be part of the structure of cyclothiopeptide gombamide A, which possess moderate inhibitory activity against Na^+^/K^+^-ATPase [22]. Further, thiazoline-based proline containing doubly-prenylated cyclopeptides like trunkamide A contain reverse prenylated ethers of serine and threonine together in their composition. Heterocyclic amino acids like histidine and tryptophan also form part of the structures of proline-rich cyclic peptides, such as wainunuamide, phakellistatin 15, 17 and stylissatin B [23,25,97]. Moreover, cytotoxic phakellistatin 3 and isophakellistatin 3 represent a new class of proline-rich cycloheptapeptides containing an unusual amino acid unit “Hpi” that apparently derived from a photooxidation product of tryptophan [100].

Moreover, five-residue cystine-linked cyclic peptides like eudistomides A, B are flanked by a *C*-terminal methyl ester and a 12-oxo- or 12-hydroxy-tetradecanoyl moiety [42]. The structure of proline containing cytotoxic peptide scleritodermin A incorporates a novel conjugated thiazole moiety 2-(1-amino-2-*p*-hydroxyphenylethane)-4-(4-carboxy-2,4-dimethyl-2*Z*,4*E*-propadiene)-thiazole (ACT) and unusual amino acids *O*-methyl-*N*-sulfo-d-serine, keto-*allo*-isoleucine [26]. The proline unit may be part of a cyclic peptide and/or may be part of a side chain, e.g., scleritodermin A, didemnin B, C and plitidepsin [26,43,45], or may be part of a linear peptide, e.g., dolastatin 15 and koshikamide A_1_ [1,5]. The methyloxazoline ring is the part of the composition of cyclohexapeptides ceratospongamides [10]. In addition, trichormamide A contains β-amino acid residue viz. β-aminodecanoic acid, in addition to two d-amino acid residues (d-Tyr and d-Leu) [49]. The wewakpeptins, proline-rich cyclic depsipeptides contain unusual moieties, like “Dhoya”, “Maba” and “Hiva” [46], and prenylagaramides B and C contain a rare “Ptyr” unit. Moreover, nostophycin bears a novel β-amino acid moiety “Ahoa” in its structure [50]. Macrocyclic depsipeptides, homodolastatin 16 and dolastatin 16 contain the new and unusual amino acid units “Pval” and “Admpa” [51,52]. Besides this, structural features for pahayokolides A and B include a pendant *N*-acetyl-*N*-methylleucine, both *E*- and *Z*-dehydrobutyrines, a homophenylalanine and an unusual polyhydroxy amino acid [48]. Oxazole and methyloxazole rings were found to be part of the structures of cyclopolypeptides myriastramides A–C and haliclonamide A [28,93], whereas *N*,*O*-dimethyl tyrosine and “Hiv” moieties were found in the structures of cytotoxic depsipeptides, tamandarins A and B [44]. The presence of two dimethylallyl threonines (or one threonine and one serine) side chains and one thiazoline ring in the backbone of the patellins is the most important feature of these compounds termed as “cyanobactins”, which have sparked attention due to their interesting bioactivities and for their potential to be prospective candidates in the development of drugs [101,102].

### 2.2. Stereochemical Aspects

**Figure 4:**
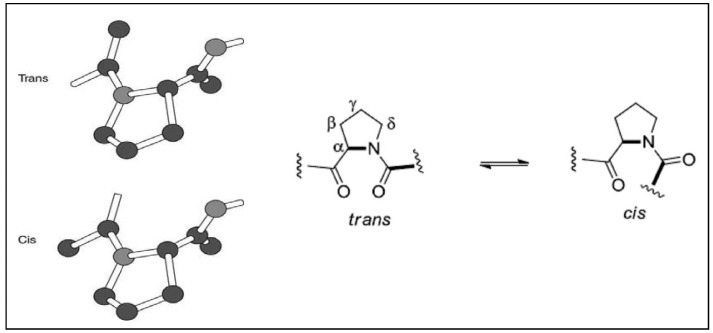
The two possible conformations for the proline peptide bond.

Structurally, proline is the only unusual amino acid with a secondary amino group based on a pyrrolidine, which forms a ring structure with rigid conformation and a secondary amine compared to the other twenty natural amino acids. This significantly reduces the structural flexibility of the polypeptide chain, and the nitrogen in the pyrrolidine ring cannot participate in hydrogen bonding with other residues [103]. Many biologically-important cyclic peptide sequences and natural products contain multiple proline residues. As seen previously for peptide bonds, the proline amide bond can also exist in *trans* or *cis* conformations (Figure 4). Peptide bonds to proline, and to other *N*-substituted amino acids, are able to populate both the *cis* and *trans* isomers. Most peptide bonds overwhelmingly adopt the *trans* isomer (typically 99.9% under unstrained conditions), because the amide hydrogen (*trans* isomer) offers less steric repulsion to the preceding C_α_ atom than does the following C_α_ atom (*cis* isomer). By contrast, the *cis* and *trans* isomers of the X-Pro peptide bond (where X represents any amino acid) both experience steric clashes with the neighboring substitution and are nearly equal energetically. Hence, the fraction of X-Pro peptide bonds in the *cis* isomer under unstrained conditions ranges from 10% to 40%; the fraction depends slightly on the preceding amino acid, with aromatic residues favoring the *cis* isomer slightly. Proline *cis*-*trans* isomerization plays a key role in the rate-determining steps of protein folding [104]. Furthermore, proline *cis*-*trans* isomerization controls autoinhibition of a signaling protein [105].

**Figure 5:**
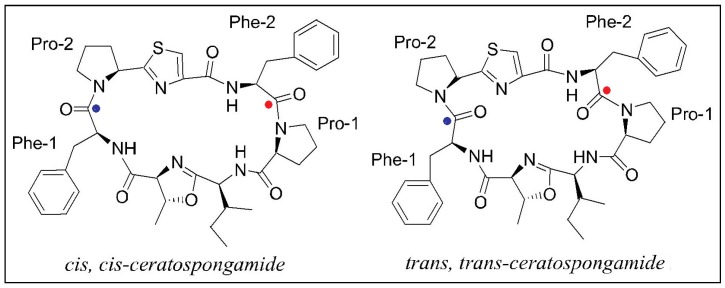
Different conformers of cyclopolypeptide ceratospongamide.

Although the *trans* amide bond is more common, the occurrence of *cis* geometry is more frequent for the proline peptide bond than for other amino acids. The frequency of the *cis* proline peptide bond is higher in cyclic peptides than in linear peptides. As per a statistical study performed on the Cambridge Structural Database, 57.4% of proline residues present in cyclic peptides were in the *cis* conformation as compared to only 5.6% in acyclic peptides [106]. The reason for this high proportion of *cis* proline in cyclopeptides is due to the conformational restrictions during the cyclisation step. The geometry of the proline amide can be determined on the basis of the difference in ^13^C chemical shifts between Cβ and Cγ signals (Δδβγ = δβ − δγ). A small ^13^C chemical shift difference indicates that the proline peptide bond is *trans*, while a large ^13^C chemical shift difference indicates a *cis* proline residue. The change in conformation of a cyclopolypeptide from “*trans*” to “*cis*” can result in loss of activity [10], e.g., the *trans*, *trans*-isomer of cyclic heptapeptide ceratospongamide showed potent inhibition of sPLA_2_ expression in a cell-based model for anti-inflammation, whereas the *cis*, *cis*-isomer was inactive (Figure 5). The distribution of the peptide bond angle omega for peptidyl-prolyl bonds in proteins shows significant peaks at 180° (*trans* peptide bond) and 0° (*cis* peptide bond). Investigations on “peptidyl-prolyl bonds and secondary structure” showed that *trans* petidyl-prolyl bonds are distributed in all types of secondary structure, whereas *cis* peptidyl is found primarily in bends and turns, suggesting a specific structural role for this type of bonding.

**Figure 6:**
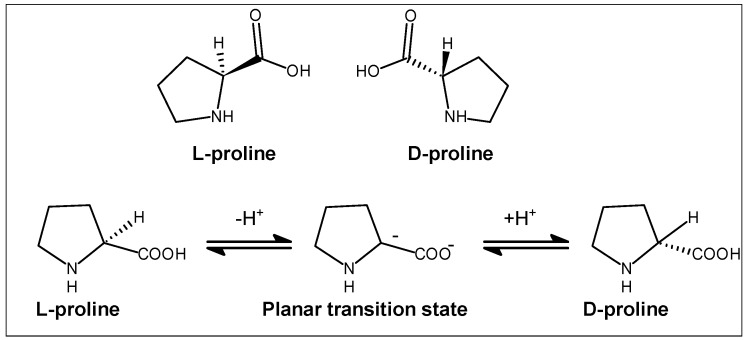
General structures of l- and d-proline and their isomerization via proline racemase.

Most amino acids occur in two possible optical isomers, called d and l (Figure 6). The l-amino acids represent the vast majority of amino acids found in proteins. l-proline is a natural non-essential amino acid, and d-proline is an unnatural amino acid, with one basic and one acidic center each. In proline, only the l-stereoisomer is involved in the synthesis of mammalian peptides/proteins.

The racemization of l-proline to d-proline proceeds through a planar transition state, where the tetrahedral α-carbon becomes trigonal as a proton leaves the l-proline. The transition-state analog for this step is pyrrolidin-2-ide-2-carboxylate (2^−^). The absolute configuration of proline residue can be determined by Marfey’s method using reagent 1-fluoro-2,4-dinitrophenyl-5-l-alanineamide (FDAA) [107]. The absolute configuration of amino proline was determined by comparing the retention time with the standard FDAA-derivatized amino acids, e.g., the structure of cyclooctapeptide reniochalistatin E contains three l-proline units with *trans* conformation [20] whereas the structure of cycloheptapeptide euryjanicin E contains three l-proline units with *cis* conformation [88]. Further, a novel cyclic tetrapeptide isolated from a *Pseudomonas* sp. (strain IM-1) associated with the marine sponge *Ircinia muscarum* was found to contain two proline units, one with l-configuration and the other with d-configuration [77].

### 2.3. Steric and Lipophilicity Parameters

In order to describe the intermolecular forces of drug receptor interaction, as well as the transport and distribution of drugs in a quantitative manner, various steric and lipophilicity parameters, like molar refractivity (MR^20^), molar volume (MV^20^), parachor (P_r_), index of refraction (n^20^), surface tension (γ^20^), density (d^20^), polarizability (α), etc., need to be calculated for natural cyclic peptides. Diverse parameters were calculated for proline-rich cyclopolypeptides of marine origin using ACD/ChemSketch software (Version 2.0, Toronto, ON, Canada) (Table S1, Supplementary Materials).

### 2.4. Synthetic Methodologies

Many proline-rich cyclic peptides were synthesized successfully by various research groups employing different techniques of peptide synthesis. The literature is enriched with reports explaining the synthesis of euryjanicin A [108], delavayin C [109], cherimolacyclopeptide G [110], psammosilenin A [111], hymenamide E [112], stylisin 1 [113], stylisin 2 [114], hymenistatin and yunnanin F [115], pseudostellarin B [116], segetalin E [117], rolloamide B [118] and pseudostellarin G [119] using the solution-phase method utilizing different carbodiimides as coupling agents, TEA/NMM as the base and the synthesis of euryjanicin B [120], mollamide [121], met-cherimolacyclopeptide B [122], axinellin A [123], phakellistatin 7 [124], phakellistatin 12 [125], petriellin A [126], hymenamide C [127], gombamide A [128] and scleritodermin A [129] by the solid-phase method of peptide synthesis. Solid-phase peptide synthesis (SPPS) results in high yields of pure products and works more quickly than classical synthesis, i.e., liquid-phase peptide synthesis (LPPS). Through the replacement of a complicated isolation procedure for each intermediate product with a simple washing procedure, much time is saved using SPPS. In addition, SPPS has proven possible to increase the yield in each individual step to 99.5% or better, which cannot be attained using conventional synthetic approaches. However, solution phase synthesis continues to be especially valuable for large-scale manufacturing and for specialized laboratory applications [130,131]. Moreover, in some cases, a mixed solid-phase/solution synthesis strategy is employed to accomplish total synthesis of the cyclopolypeptide [132], e.g., during the total synthesis of the naturally-occurring proline-rich cyclic octapeptide stylissamide X, the linear octapeptide was assembled first by standard Fmoc solid-phase peptide synthesis (SPPS), and cyclization was carried out subsequently by the solution method. Total synthesis can also be achieved via a convergent native chemical ligation-oxidation strategy [133], e.g., polydiscamides B–D, or utilizing diethyl phosphorocyanidate/BOP-Cl chemistry [134], e.g., axinastatins 2 and 3.

## 3. Biological Status

l-proline itself is an osmoprotectant and is used in many pharmaceutical and biotechnological applications, whereas the proline analogue *cis*-4-hydroxy-l-proline has been clinically evaluated as an anticancer drug. Although proline-rich cyclopolypeptides of marine origin are associated with a number of bioactivities, including anti-cancer, anti-tuberculosis, anti-inflammatory, anti-viral, immunosuppressive and anti-fungal activities, still the majority of them were found to exhibit cell growth inhibitory activity [135,136]. Various pharmacological activities associated marine-derived proline-rich cyclopeptides along with susceptible cell line/organism with minimum inhibitory concentration are compiled in Table 2.

### 3.1. Mechanism of Action

In drug development, a good antimicrobial candidate should exhibit highly specific biological activity followed by a good pharmacokinetic profile and low immunogenicity. Studies have demonstrated that the members of the proline-rich peptide group and their derivatives act with a completely divergent mechanism than the lytic amphiphilic antimicrobial peptides. Retaining highly potent antimicrobial activities, proline-rich antimicrobial peptides subsequently act in a divergent way, including stereospecific interaction with the membrane translocation system followed by intracellular targeting, compared with the more general membrane disruption mode of action of traditional antimicrobial peptides. It has been further suggested that proline-rich antimicrobial peptides stereo-specifically bind to intracellular targets, such as the bacterial heat shock DnaK protein, and this binding can be correlated with the observed antimicrobial activity. Moreover, proline-rich peptides are characterized by good water solubility, high potency against bacteria killing and low cytotoxic effects at high concentrations, making them attractive lead candidates for the development of novel antimicrobial therapeutic agents [103].

Further, proline-rich antimicrobial peptides are actively transported inside the bacterial cell where they bind and inactivate specific targets like the bacterial ribosome and, thereby, inhibit protein synthesis. This implies that they can be used as molecular hooks to identify the intracellular or membrane proteins that are involved in their mechanism of action and that may be subsequently used as targets for the design of novel antibiotics with mechanisms different from those now in use. Didemnin B is a heterodetic non-polar cyclic peptide associated with antiviral, antitumor, immunomodulating properties, potently inhibits protein and DNA synthesis by binding to the eukaryotic translation elongation factor EF-1α in a GTP-dependent manner, and the formation of the didemnin B-GTP-EF-1α complex may be responsible for the observed inhibition of protein synthesis [139]. Inhibition of protein synthesis by didemnin B occurs by stabilization of aminoacyl-tRNA to the ribosomal A-site, preventing the translocation of phenylalanyl-tRNA from the A- to the P-site, but not preventing peptide bond formation. Tamandarin A may act by the same mechanism as didemnin B. Aplidine’s (dehydrodidemnin B) mechanism of action involves several pathways, including cell cycle arrest and inhibition of protein synthesis. Aplidine induces early oxidative stress and results in a rapid and persistent activation of JNK and p38 MAPK phosphorylation with activation of both kinases occurring very rapid, long before the execution of apoptosis [140]. Didemnin B induces the death of a variety of transformed cells with apoptotic morphology, DNA fragmentation within the cytosol and the generation of DNA ladders. Scleritodermin A acts by tubulin polymerization inhibition [26].

The immunosuppressive activity of cyclolinopeptide A results from the formation of the complex with cyclophilin and inhibition of the phosphatase activity of calcineurin, a phosphatase that plays an important role in T lymphocyte signaling [141]. Cemadotin (LU103793) is a water-soluble synthetic analogue of linear peptide dolastatin 15, which is believed to act on microtubules involving binding to tubulin and strong suppression of microtubule dynamics.

### 3.2. Peptide Market and PRCPs in Clinical Trials

Currently, there are more than 60 U.S. Food and Drug Administration (FDA)-approved peptide medicines on the market, and this is expected to grow significantly, with approximately 140 peptide drugs currently in clinical trials and more than 500 therapeutic peptides in preclinical development. In terms of value, the global peptide drug market has been predicted to increase from US$14.1 billion in 2011 to an estimated US$25.4 billion in 2018, with an underlying increase in novel innovative peptide drugs from US$8.6 billion in 2011 (60%) to US$17.0 billion (66%) in 2018 [74]. Currently, most peptide drugs are administered by the parental route, and approximately 75% are given as injectables. However, alternative administration forms are gaining increasing traction, including oral, intranasal and transdermal delivery routes, according to the respective technology developments. The use of alternative administration forms could also enable greater usage of peptide therapeutics in other disease areas, such as inflammation, where topical administration of peptides could be the basis for highly efficacious novel treatments.

The cyclic depsipeptide didemnin B was the first marine-derived cyclopolypeptide to undergo clinical trials targeted at oncological patients. However, high toxicity, poor solubility and short life span led to the discontinuation of clinical trials of didemnin B and rendered it unsuitable for further drug development [142]. The linear depsipeptide kahalalide F is known for its antifungal and antitumor activities, and its phase II clinical trials are underway. Another cyclic depsipeptide plitidepsin (dehydrodidemnin B or aplidine) is in clinical development. In 2003, plitidepsin was granted orphan drug status by the European Medicines Agency for treating acute lymphoblastic leukemia. In 2007, it was undergoing multicenter phase II clinical trials, and in 2016, early results in a small phase I trial for multiple myeloma were announced. The two most promising peptides of antimitotic dolastatins group, dolastatin 10 and 15, were selected for development and are currently undergoing phase II clinical trials. Cemadotin, the synthetic analogue of dolastatin 15, is also in phase II clinical trials as a promising cancer chemotherapeutic agent [143,144].

## 4. Conclusions and Future Prospects

There is increased evidence of the emergence of resistance to conventional drugs illustrating the importance of research on natural peptide-based drug development. PRCPs have several structural features making them good drug leads, and there are several naturally-occurring cyclic peptides in clinical use and in clinical trials. In addition, biologically-active proline-rich cyclic peptides have been developed with synthetic approaches, and they are useful as therapeutics and biochemical tools. With the introduction of new high throughput screening methods, there will be more availability of marine-based PRCPs with interesting biological properties. PRCPs can work on their targets very selectively, as the interaction with the targets is very specific compared to small molecules. In addition to the merits of peptides, especially “proline-rich cyclic structures” as drug molecules, cyclopolypeptides could make even better peptide drugs for future use. Moreover, the future development of peptide drugs will continue to build upon the strengths of naturally-occurring proline-rich peptides, with the application of traditional rational design to improve their weaknesses, such as their chemical and physical properties. Further, emerging peptide technologies will help broaden the applicability of PRCPs as therapeutics. While still in the early stages of development, PRCPs drug leads have started gaining the attention of the pharmaceutical industry; however, their true potential is still very much unknown.

## Figures and Tables

**Table 1:** Proline-rich cyclopolypeptides from marine resources.

Year	Cyclic Peptide	Molecular Formula	No. of Proline Units	Composition
1981	Didemnin B [43]	C_57_H_89_N_7_O_15_	two	cyclodepsipeptide
1988	Aplidine [45]	C_57_H_87_N_7_O_15_		cyclodepsipeptide
1991	Axinastatin 1 [6]	C_38_H_56_N_8_O_8_		cycloheptapeptide
1992	Malaysiatin [27]	C_38_H_56_N_8_O_8_		cycloheptapeptide
1992	Polydiscamide A [7]	C_76_H_109_BrN_19_O_20_SNa		cyclodepsipeptide
1993	Axinastatin 4 [76]	C_42_H_62_N_8_O_8_		cycloheptapeptide
1993	Cyclooligopeptide [77]	C_24_H_32_N_4_O_5_		cyclotetrapeptide
1993	Hymenamide B [31]	C_43_H_56_N_8_O_10_		cycloheptapeptide
1993	Hymenamide C [8]	C_43_H_54_N_8_O_9_		cycloheptapeptide
1993	Hymenamide D [8]	C_38_H_55_N_7_O_10_		cycloheptapeptide
1993	Hymenamide E [8]	C_45_H_55_N_7_O_10_		cycloheptapeptide
1994	Mollamide [40]	C_42_H_61_N_7_O_7_S		cycloheptapeptide
1994	Schizotrin A [78]	C_72_H_107_N_13_O_21_		cycloundecapeptide
1994	Axinastatin 2 [39]	C_39_H_58_N_8_O_8_		cycloheptapeptide
1994	Axinastatin 3 [39]	C_40_H_61_N_8_O_8_		cycloheptapeptide
1995	Stylopeptide 1 [79]	C_40_H_61_N_7_O_8_		cycloheptapeptide
1996	Patellin 3 [80]	C_48_H_78_N_8_O_9_S		cyclooctapeptide
1996	Patellin 4 [80]	C_47_H_76_N_8_O_9_S		cyclooctapeptide
1996	Patellin 5 [80]	C_49_H_72_N_8_O_9_S		cyclooctapeptide
1996	Patellin 6 [80]	C_50_H_74_N_8_O_9_S		cyclooctapeptide
1996	Hymenamide F [81]	C_35_H_60_N_10_O_7_S		cycloheptapeptide
1996	Agardhipeptin B [82]	C_57_H_69_N_11_O_8_		cyclooctapeptide
1996	Kapakahine A [37]	C_58_H_72_N_10_O_9_		cyclooctapeptide
1996	Kapakahine C [37]	C_58_H_72_N_10_O_10_		cyclooctapeptide
1996	Kapakahine D [37]	C_58_H_72_N_10_O_10_		cyclooctapeptide
1998	Axinellin A [21]	C_42_H_56_N_8_O_9_		cycloheptapeptide
1998	Shearamide A [83]	C_47_H_63_N_9_O_9_		cyclooctapeptide
1999	Prenylagaramide B [84]	C_49_H_68_N_8_O_10_		cycloheptapeptide
1999	Nostophycin [50]	C_46_H_64_N_8_O_10_		cycloheptapeptide
2000	*trans,trans*-ceratospongamide [10]	C_41_H_49_N_7_O_6_S		cycloheptapeptide
2000	Tamandarine A [44]	C_54_H_87_N_7_O_14_		cyclodepsipeptide
2000	Tamandarine B [44]	C_53_H_82_N_7_O_14_		cyclodepsipeptide
2001	Microspinosamide [38]	C_75_H_109_BrN_18_O_22_S		cyclodepsipeptide
2003	Myriastramide C [28]	C_42_H_53_N_9_O_7_S		cyclooctapeptide
2004	Scleritodermin A [26]	C_42_H_54_N_7_O_10_SNa		cyclodepsipeptide
2004	Cyclonellin [85]	C_45_H_62_N_12_O_12_		cyclooctapeptide
2005	Wewakpeptin A [46]	C_52_H_85_N_7_O_11_		cyclodepsipeptide
2005	Wewakpeptin B [46]	C_52_H_89_N_7_O_11_		cyclodepsipeptide
2005	Wewakpeptin C [46]	C_54_H_81_N_7_O_11_		cyclodepsipeptide
2005	Wewakpeptin D [46]	C_54_H_85_N_7_O_11_		cyclodepsipeptide
2007	Pahayokolide A [48]	C_72_H_105_N_13_O_20_		cycloundecapeptide
2007	Pahayokolide B [48]	C_63_H_90_N_12_O_18_		cycloundecapeptide
2008	Polydiscamide B [18]	C_75_H_110_BrN_18_O_21_S		cyclodepsipeptide
2008	Polydiscamide C [18]	C_74_H_107_BrN_18_O_21_S		cyclodepsipeptide
2008	Polydiscamide D [18]	C_73_H_105_BrN_18_O_21_S		cyclodepsipeptide
2009	Euryjanicin A [36]	C_44_H_58_N_8_O_8_		cycloheptapeptide
2009	Euryjanicin C [14]	C_40_H_61_N_7_O_8_		cycloheptapeptide
2009	Euryjanicin D [14]	C_44_H_59_N_7_O_8_		cycloheptapeptide
2009	Eudistomide A [42]	C_37_H_61_N_5_O_8_S_2_		cyclolipopeptide
2009	Eudistomide B [42]	C_37_H_63_N_5_O_8_S_2_		cyclolipopeptide
2010	Anacyclamide A10 [86]	C_49_H_72_N_12_O_14_		cyclodecapeptide
2011	Duanbanhuain A [87]	C_43_H_58_N_8_O_11_		cyclooctapeptide
2011	Duanbanhuain B [87]	C_45_H_57_N_9_O_10_		cyclooctapeptide
2012	Mollamide F [12]	C_33_H_46_N_6_O_5_S		cyclohexapeptide
2013	Stylissatin A [24]	C_49_H_63_N_7_O_8_		cycloheptapeptide
2013	Euryjanicin E [88]	C_44_H_60_N_8_O_8_		cycloheptapeptide
2013	Euryjanicin F [88]	C_49_H_63_N_7_O_7_		cycloheptapeptide
2013	Gombamide A [22]	C_38_H_45_N_7_O_8_S_2_		cyclothiohexapeptide
2013	Cycloforskamide [41]	C_54_H_86_N_12_O_11_S_3_		cyclododecapeptide
2014	Trichormamide A [49]	C_58_H_93_N_11_O_15_		cycloundecapeptide
2014	Reniochalistatin A [20]	C_37_H_62_N_8_O_8_		cycloheptapeptide
2016	Carteritin B [34]	C_46_H_57_N_7_O_11_		cycloheptapeptide
1990	Hymenistatin 1 [35]	C_47_H_72_N_8_O_9_	three	cyclooctapeptide
1993	Phakellistatin 1 [32]	C_45_H_61_N_7_O_8_		cycloheptapeptide
1993	Hymenamide A [31]	C_46_H_61_N_11_O_7_		cycloheptapeptide
1993	Phakellistatin 2 [89]	C_45_H_61_N_7_O_8_		cycloheptapeptide
1994	Axinastatin 5 [30]	C_47_H_72_N_8_O_9_		cyclooctapeptide
1994	Hymenamide G [90]	C_47_H_72_N_8_O_9_		cyclooctapeptide
1994	Hymenamide H [90]	C_47_H_69_N_9_O_9_		cyclooctapeptide
1995	Phakellistatin 11 [91]	C_53_H_67_N_9_O_9_		cyclooctapeptide
1996	Waiakeamide [12]	C_37_H_49_N_7_O_8_S_3_		cyclohexapeptide
1998	Axinellin B [21]	C_50_H_67_N_9_O_9_		cyclooctapeptide
2000	Haligramide A [92]	C_37_H_49_N_7_O_6_S_3_		cyclohexapeptide
2000	Haligramide B [92]	C_37_H_49_N_7_O_7_S_3_		cyclohexapeptide
2001	Haliclonamide A [93]	C_45_H_60_N_8_O_9_		cyclooctapeptide
2001	Haliclonamide B [93]	C_40_H_52_N_8_O_9_		cyclooctapeptide
2001	Wainunuamide [23]	C_38_H_51_N_9_O_7_		cycloheptapeptide
2002	Axinellin C [94]	C_50_H_67_N_9_O_9_		cyclooctapeptide
2002	Dolastatin 16 [52]	C_47_H_70_N_6_O_10_		cyclodepsipeptide
2002	Haliclonamide C [95]	C_45_H_60_N_8_O_10_		cyclooctapeptide
2002	Haliclonamide D [95]	C_40_H_54_N_8_O_10_		cyclooctapeptide
2002	Haliclonamide E [95]	C_45_H_62_N_8_O_10_		cyclooctapeptide
2003	Myriastramide A [28]	C_45_H_58_N_8_O_9_		cyclooctapeptide
2003	Myriastramide B [28]	C_45_H_57_ClN_8_O_9_		cyclooctapeptide
2003	Wewakazole [96]	C_59_H_72_N_12_O_12_		cyclododecapeptide
2005	Dominicin [16]	C_43_H_72_N_8_O_9_		cyclooctapeptide
2006	Stylisin 1 [19]	C_45_H_61_N_7_O_8_		cycloheptapeptide
2009	Euryjanicin B [14]	C_36_H_51_N_7_O_8_		cycloheptapeptide
2010	Phakellistatin 15 [25]	C_48_H_71_N_9_O_9_		cyclooctapeptide
2010	Phakellistatin 17 [25]	C_49_H_73_N_9_O_8_		cyclooctapeptide
2010	Phakellistatin 18 [25]	C_45_H_61_N_7_O_8_		cycloheptapeptide
2010	Callyaerin B [13]	C_65_H_108_N_12_O_14_		cyclooctapeptide ^b^
2010	Callyaerin C [13]	C_70_H_105_N_13_O_16_		cycloheptapeptide ^c^
2012	Stylissamide X [33]	C_51_H_69_N_9_O_9_		cyclooctapeptide
2013	Euryjanicin G [88]	C_48_H_59_N_7_O_7_		cyclooctapeptide
2014	Reniochalistatins E [20]	C_49_H_73_N_9_O_8_		cyclooctapeptide
2016	Carteritin A [34]	C_44_H_57_N_7_O_10_		cycloheptapeptide
2016	Stylissatin B [97]	C_38_H_51_N_9_O_7_		cycloheptapeptide
2016	Stylissatin C [97]	C_39_H_55_N_7_O_9_		cycloheptapeptide
2016	Stylissatin D [97]	C_40_H_57_N_7_O_9_		cycloheptapeptide
2016	Wewakazole B [47]	C_58_H_70_N_12_O_12_		cyclododecapeptide
1968	Antamanide [98]	C_64_H_78_N_10_O_10_	four	cyclodecapeptide
2004	Callynormine A [15]	C_61_H_93_N_11_O_13_		cycloheptapeptide ^b^
2006	Stylisin 2 [19]	C_44_H_57_N_7_O_8_		cycloheptapeptide
2008	Stylopeptide 2 [29]	C_63_H_84_N_10_O_12_		cyclodecapeptide
2010	Callyaerin A [13]	C_69_H_108_N_14_O_14_		cyclooctapeptide ^c^
2010	Callyaerin E [13]	C_66_H_94_N_12_O_13_		cycloheptapeptide ^c^
2010	Callyaerin H [13]	C_54_H_81_N_11_O_10_		cycloheptapeptide ^a^
2008	Callyaerin G [99]	C_69_H_91_N_13_O_12_	five	cycloheptapeptide ^c^

With ^a^ dipeptide, ^b^ tripeptide and ^c^ tetrapeptide side chains.

**Table 2:** Marine-derived proline-rich cyclopeptides with diverse bioactivities.

PRCPs	Resource	Pharmacological Activity	
		Susceptibility	MIC Value
Axinastatin 1 [6]	marine sponge	Cytotoxicity against PS leukemia cell line	0.21 μg/mL
Polydiscamide A [7]	marine sponge	Antiproliferative activity against human lung cancer A549 cell line; antibacterial activity against *Bacillus subtilis*	0.7 μg/mL; 3.1 μg/mL
Hymenamide E [8]	marine sponge	Antifungal activity against pathogenic *Cryptococcus neoformans*	133 μg/mL
*trans*,*trans*-Ceratospongamide [10]	marine red alga	Inhibition of sPLA_2_ expression in a cell-based model for anti-inflammation	0.0013 μg/mL
Mollamide F [12]	marine tunicate	Anti-HIV activity in cytoprotective cell-based assay and HIV integrase inhibition assay	0.0016 and 0.0031 μg/mL
Callyaerin A [13]	marine sponge	Anti-TB activity against *M. tuberculosis*, inhibitory activity toward *C. albicans*	7.37 μg/mL
Callyaerin B [13]	marine sponge	Anti-TB activity against *Mycobacterium tuberculosis*	7.8 μg/mL
Callyaerin E, H [13]	marine sponge	Cytotoxicity against L5178Y cell line	7.91 and 9.59 μg/mL
Euryjanicin C [14]	marine sponge	Inhibitory activity against human hepatitis B virus	49 μg/mL
Polydiscamides B–D [18]	marine sponge	Agonist activity against human sensory neuron-specific G protein couple receptor (SNSR) that is involved in the modulation of pain	-
Axinellin A, B [21]	marine sponge	Antitumor activity against human bronchopulmonary non-small-cell lung-carcinoma lines (NSCLC-N6)	3.0 and 7.3 μg/mL
Wainunuamide [23]	marine sponge	Cytotoxic activity against A2780 ovarian tumor and K562 leukemia cancer cells	19.15 and 18.36 μg/mL
Stylissatin A [24]	marine sponge	Inhibition of NO production in LPS-stimulated RAW264.7 cells	0.0011 μg/mL
Scleritodermin A [26]	marine sponge	Inhibition of tubulin polymerization and human tumor cell lines	-
Axinastatin 5 [30]	marine sponge	Cytotoxic activity against human and murine cancer cells	0.3–3.3 μg/mL
Phakellistatin 1 [32]	marine sponges	Cell growth inhibitory activity against P-388 murine leukemia	7.5 μg/mL
Stylissamide X [33]	marine sponge	Inhibitory activity against migration of HeLa cells	0.001–0.1 μg/mL
Carteritin A [34]	marine sponge	Cytotoxicity against HeLa, HCT116 and RAW264 cells	0.0012–0.0026 μg/mL
Hymenistatin 1 [35]	marine sponge	Cytotoxicity against P-388 leukemia cells	3.5 μg/mL
Kapakahine A, C [37]	marine sponge	Cytotoxicity against P-388 murine leukemia cells	5.4 and 5.0 μg/mL
Microspinosamide [38]	marine sponge	Anti-HIV activity in CEM-SS cells	0.2 μg/mL
Axinastatin 2 [39]	marine sponge	Cytotoxicity against murine leukemia P-388 cell line	0.02 μg/mL
Axinastatin 3 [39]	marine sponge	Cytotoxicity against PS leukemia cell line	0.4 μg/mL
Mollamide [40]	sea squirt	Cytotoxicity against P-388 (murine leukemia) and A549 (human lung carcinoma), HT29 (human colon carcinoma) cells	1.0–2.5 μg/mL
Cycloforskamide [41]	sea slug	Cytotoxicity against murine leukemia P-388 cells	8.51 μg/mL
Didemnin B [43]	marine tunicate	Cytotoxic activity against human L1210 lymphocytic leukemia cell lines; pancreatic carcinoma (BX-PC3) cell lines; prostatic cancer (DU-145) cell lines; head and neck carcinoma (UMSCC10b) cell lines	0.0025 μg/mL; 0.002 μg/mL; 0.0015 μg/mL; 0.0018 μg/mL
Tamandarin A [44]	marine ascidian	Cytotoxic activity against human pancreatic carcinoma (BX-PC3) cell lines; prostatic cancer (DU-145) cell lines; head and neck carcinoma (UMSCC10b) cell lines	0.0018 μg/mL; 0.0014 μg/mL; 0.0009 μg/mL
Wewakpeptin A [46]	marine cyanobacterium	Cytotoxicity against NCI-H460 human lung tumor and the neuro-2a mouse neuroblastoma cell lines	0.001 μg/mL
Wewakazole B [47]	marine cyanobacterium	Cytotoxicity against human MCF7 breast/H460 lung cancer cells	8.87–15.29 μg/mL
Pahayokolide A [48]	marine cyanobacteria	Antibacterial activity against *Bacillus megaterium*, *Bacillus subtilis*	5 μg/mL
Trichormamide A [49]	marine cyanobacteria	Antiproliferative activities against the human melanoma cell line (MDA-MB-435) and the human colon cancer cell line (HT-29)	8.45 and 8.53 μg/mL
Axinastatin 4 [76]	marine sponge	Cytotoxic activity against P-388 lymphocytic leukemia cell line	0.057 μg/mL
Phakellistatin 2 [89]	marine sponge	Cell growth inhibitory activity against P-388 cell line	0.34 μg/mL
Phakellistatin 7–9 [137]	marine sponge	Cell growth inhibitory activity against P-388 murine leukemia	3.0, 2.9 and 4.1 μg/mL
Axinellin C [94]	marine sponge	Cytotoxic activity against A2780 ovarian tumor and K562 leukemia cancer cells	13.17 and 4.46 μg/mL
Callyaerin G [99]	marine sponge	Cytotoxic towards the mouse lymphoma cell line (L5178Y) and HeLa cells	0.53 and 5.4 μg/mL
Stylissatin B [97]	marine sponge	Inhibitory effects against human tumor cell lines including HCT-116, HepG2, BGC-823, NCI-H1650, A2780 and MCF7	0.0013 μg/mL
Phakellistatin 10, 11 [91]	marine sponge	Cell growth inhibitory activity against murine P-388 lymphocytic leukemia	2.1, 0.20 μg/mL
Stylopeptide 1 [79]	marine sponge	Cell growth inhibitory activity against murine P-388 lymphocytic leukemia	0.01 μg/mL
Phakellistatin 12 [138]	marine sponge	Cell growth inhibitory activity against murine P-388 lymphocytic leukemia	2.8 μg/mL

## Supplementary Materials

The following are available online at www.mdpi.com/1660-3397/14/11/194/s1. Table S1: Various steric and lipophilic parameters for proline-rich cyclopolypeptides from diverse marine resources.
