# Supplementary material for: Natural Proline-Rich Cyclopolypeptides from Marine Organisms: Chemistry, Synthetic Methodologies and Biological Status
Source: Mar Drugs. 2016 Oct 26;14(11):194. doi: 10.3390/md14110194 (PMC5128737; doi:10.3390/md14110194)
Supplement: Supplementary file 1 [file marinedrugs-14-00194-s001.docx]

Supplementary Materials: Natural Proline-Rich Cyclopolypeptides from Marine Organisms: Chemistry, Synthetic Methodologies and Biological Status

Wan-Yin Fang, Rajiv Dahiya, Hua-Li Qin, Rita Mourya and Sandeep Maharaj

**Table S1.** Various steric and lipophilic parameters for proline-rich cyclopolypeptides from diverse marine resources.

| **Cyclic Peptide** | **Mol. wt.** | **MR^20^ (cm^3^)** | **MV^20^ (cm^3^)** | **P_r_ (cm^3^)** | **n^20^** | **γ^20^ (Dyne/cm)** | **d^20^ (g/cm^3^)** | **α (10^−24^ cm^3^)** |
| --- | --- | --- | --- | --- | --- | --- | --- | --- |
| Malaysiatin [1] | 752.91 | 199.15±0.4 | 586.1 ± 5.0 | 1642.1 ± 6.0 | 1.595 ± 0.03 | 61.6 ± 5.0 | 1.28 ± 0.1 | 78.95 ± 0.5 |
| Stylopeptide 2 [2] | 1173.41 | 315.72 ± 0.4 | 875.6 ± 5.0 | 2539.4 ± 6.0 | 1.640 ± 0.03 | 70.7 ± 5.0 | 1.34 ± 0.1 | 125.16 ± 0.5 |
| Carteritin A [3] | 843.97 | 221.55 ± 0.4 | 613.0 ± 5.0 | 1791.1 ± 6.0 | 1.642 ± 0.03 | 72.8 ± 5.0 | 1.37 ± 0.1 | 87.82 ± 0.5 |
| Carteritin B [3] | 883.99 | 232.49 ± 0.4 | 623.3 ± 5.0 | 1861.3 ± 6.0 | 1.668 ± 0.03 | 79.4 ± 5.0 | 1.41 ± 0.1 | 92.16 ± 0.5 |
| Stylissamide X [4] | 952.16 | 258.23 ± 0.4 | 717.4 ± 5.0 | 2064.6 ± 6.0 | 1.639 ± 0.03 | 68.5 ± 5.0 | 1.32 ± 0.1 | 102.37 ± 0.5 |
| Reniochalistatin A [5] | 746.94 | 197.57 ± 0.4 | 608.9 ± 5.0 | 1666.5 ± 6.0 | 1.562 ± 0.03 | 56.1 ± 5.0 | 1.22 ± 0.1 | 78.32 ± 0.5 |
| Reniochalistatin E [5] | 916.17 | 250.48 ± 0.4 | 727.2 ± 5.0 | 2032.0 ± 6.0 | 1.605 ± 0.03 | 60.9 ± 5.0 | 1.25 ± 0.1 | 99.30 ± 0.5 |
| Phakellistatin 7 [6] | 1109.37 | 298.73 ± 0.4 | 853.1 ± 5.0 | 2428.6 ± 6.0 | 1.617 ± 0.03 | 65.6 ± 5.0 | 1.30 ± 0.1 | 118.42 ± 0.5 |
| Phakellistatin 1 [7] | 828.02 | 224.50 ± 0.4 | 635.8 ± 5.0 | 1806.9 ± 6.0 | 1.623 ± 0.03 | 65.2 ± 5.0 | 1.30 ± 0.1 | 89.00 ± 0.5 |
| Phakellistatin 15 [8] | 918.14 | 247.39 ± 0.4 | 708.4 ± 5.0 | 2008.9 ± 6.0 | 1.615 ± 0.03 | 64.6 ± 5.0 | 1.29 ± 0.1 | 98.07 ± 0.5 |
| Phakellistatin 17 [8] | 916.17 | 250.48 ± 0.4 | 727.2 ± 5.0 | 2032.0 ± 6.0 | 1.605 ± 0.03 | 60.9 ± 5.0 | 1.25 ± 0.1 | 99.30 ± 0.5 |
| Phakellistatin 18 [8] | 828.02 | 224.50 ± 0.4 | 635.8 ± 5.0 | 1806.9 ± 6.0 | 1.623 ± 0.03 | 65.2 ± 5.0 | 1.30 ± 0.1 | 89.00 ± 0.5 |
| Duanbanhuain A [9] | 862.97 | 223.89 ± 0.4 | 622.0 ± 5.0 | 1825.7 ± 6.0 | 1.639 ± 0.03 | 74.1 ± 5.0 | 1.38 ± 0.1 | 88.75 ± 0.5 |
| Duanbanhuain B [9] | 883.99 | 232.45 ± 0.4 | 636.5 ± 5.0 | 1866.9 ± 6.0 | 1.650 ± 0.03 | 73.9 ± 5.0 | 1.38 ± 0.1 | 92.15 ± 0.5 |
| Stylissatin A [10] | 878.08 | 241.76 ± 0.4 | 681.4 ± 5.0 | 1932.0 ± 6.0 | 1.627 ± 0.03 | 64.6 ± 5.0 | 1.28 ± 0.1 | 95.84 ± 0.5 |
| Stylissatin B [11] | 745.87 | 199.22 ± 0.4 | 546.9 ± 5.0 | 1592.4 ± 6.0 | 1.636 ± 0.03 | 71.8 ± 5.0 | 1.36 ± 0.1 | 77.78 ± 0.5 |
| Stylissatin C [11] | 765.90 | 199.91 ± 0.4 | 575.3 ± 5.0 | 1640.0 ± 6.0 | 1.611 ± 0.03 | 66.0 ± 5.0 | 1.33 ± 0.1 | 79.25 ± 0.5 |
| Stylissatin D [11] | 779.93 | 204.75 ± 0.4 | 600.0 ± 5.0 | 1683.4 ± 6.0 | 1.598 ± 0.03 | 61.9 ± 5.0 | 1.29 ± 0.1 | 81.16 ± 0.5 |
| Axinellin A [12] | 816.95 | 216.17 ± 0.4 | 608.3 ± 5.0 | 1754.8 ± 6.0 | 1.628 ± 0.03 | 69.2 ± 5.0 | 1.34 ± 0.1 | 85.69 ± 0.5 |
| Axinellin B [12] | 938.13 | 253.60 ± 0.4 | 701.5 ± 5.0 | 2024.5 ± 6.0 | 1.642 ± 0.03 | 69.3 ± 5.0 | 1.33 ± 0.1 | 100.53 ± 0.5 |
| Axinellin C [13] | 938.13 | 253.60 ± 0.4 | 701.5 ± 5.0 | 2024.5 ± 6.0 | 1.642 ± 0.03 | 69.3 ± 5.0 | 1.33 ± 0.1 | 100.53 ± 0.5 |
| Wainunuamide [14] | 745.87 | 196.22 ± 0.4 | 546.9 ± 5.0 | 1592.4 ± 6.0 | 1.636 ± 0.03 | 71.8 ± 5.0 | 1.36 ± 0.1 | 77.78 ± 0.5 |
| Hymenistatin 1 [15] | 893.13 | 240.53 ± 0.4 | 711.7 ± 5.0 | 1980.9 ± 6.0 | 1.591 ± 0.03 | 60.0 ± 5.0 | 1.25 ± 0.1 | 95.35 ± 0.5 |
| Euryjanicin A [16] | 826.99 | 223.91 ± 0.4 | 620.5 ± 5.0 | 1786.2 ± 6.0 | 1.641 ± 0.03 | 68.6 ± 5.0 | 1.33 ± 0.1 | 88.76 ± 0.5 |
| Euryjanicin B [17] | 709.84 | 185.90 ± 0.4 | 530.6 ± 5.0 | 1514.6 ± 6.0 | 1.617 ± 0.03 | 66.3 ± 5.0 | 1.33 ± 0.1 | 73.69 ± 0.5 |
| Euryjanicin C [17] | 767.96 | 206.21 ± 0.4 | 615.1 ± 5.0 | 1704.3 ± 6.0 | 1.585 ± 0.03 | 58.9 ± 5.0 | 1.24 ± 0.1 | 81.75 ± 0.5 |
| Euryjanicin D [17] | 801.98 | 217.06 ± 0.4 | 624.3 ± 5.0 | 1760.0 ± 6.0 | 1.612 ± 0.03 | 63.1 ± 5.0 | 1.28 ± 0.1 | 86.04 ± 0.5 |
| Dominicin [18] | 845.09 | 225.06 ± 0.4 | 687.1 ± 5.0 | 1887.0 ± 6.0 | 1.568 ± 0.03 | 56.8 ± 5.0 | 1.22 ± 0.1 | 89.22 ± 0.5 |
| Gombamide A [19] | 791.93 | 207.87 ± 0.4 | 543.9 ± 5.0 | 1626.9 ± 6.0 | 1.689 ± 0.03 | 80.0 ± 5.0 | 1.45 ± 0.1 | 82.40 ± 0.5 |
| Hymenamide B [20] | 844.96 | 220.94 ± 0.4 | 620.3 ± 5.0 | 1802.1 ± 6.0 | 1.630 ± 0.03 | 71.2 ± 5.0 | 1.36 ± 0.1 | 87.58 ± 0.5 |
| Hymenamide C [21] | 826.94 | 219.44 ± 0.4 | 600.0 ± 5.0 | 1755.4 ± 6.0 | 1.652 ± 0.03 | 73.2 ± 5.0 | 1.37 ± 0.1 | 86.99 ± 0.5 |
| Hymenamide F [22] | 764.98 | 198.94 ± 0.5 | 542.3 ± 7.0 | 1486.5 ± 8.0 | 1.654 ± 0.05 | 56.4 ± 7.0 | 1.41 ± 0.1 | 78.86 ± 0.5 |
| Axinastatin 4 [23] | 806.99 | 217.67 ± 0.4 | 628.0 ± 5.0 | 1768.5 ± 6.0 | 1.609 ± 0.03 | 62.8 ± 5.0 | 1.28 ± 0.1 | 86.29 ± 0.5 |
| Axinastatin 5 [24] | 893.13 | 240.53 ± 0.4 | 711.7 ± 5.0 | 1980.9 ± 6.0 | 1.591 ± 0.03 | 60.0 ± 5.0 | 1.25 ± 0.1 | 95.35 ± 0.5 |
| Stylisin 1 [25] | 828.02 | 224.50 ± 0.4 | 635.8 ± 5.0 | 1806.9 ± 6.0 | 1.623 ± 0.03 | 65.2 ± 5.0 | 1.30 ± 0.1 | 89.00 ± 0.5 |
| Stylisin 2 [25] | 811.97 | 218.08 ± 0.4 | 599.2 ± 5.0 | 1737.5 ± 6.0 | 1.648 ± 0.03 | 70.6 ± 5.0 | 1.35 ± 0.1 | 86.45 ± 0.5 |
| Haligramide A [26] | 784.02 | 208.08 ± 0.4 | 563.5 ± 5.0 | 1647.5 ± 6.0 | 1.660 ± 0.03 | 73.0 ± 5.0 | 1.39 ± 0.1 | 82.49 ± 0.5 |
| Haligramide B [26] | 800.01 | 208.94 ± 0.4 | 559.6 ± 5.0 | 1668.1 ± 6.0 | 1.669 ± 0.03 | 78.9 ± 5.0 | 1.42 ± 0.1 | 82.83 ± 0.5 |
| Waiakeamide [27] | 816.01 | 211.57 ± 0.4 | 575.3 ± 5.0 | 1688.2 ± 6.0 | 1.656 ± 0.03 | 74.1 ± 5.0 | 1.41 ± 0.1 | 83.87 ± 0.5 |
| Callyaerin A [28] | 1357.69 | 362.62 ± 0.4 | 1061.7 ± 5.0 | 3001.4 ± 6.0 | 1.598 ± 0.03 | 63.8 ± 5.0 | 1.27 ± 0.1 | 143.75 ± 0.5 |
| Eudistomide A [29] | 768.03 | 204.42 ± 0.4 | 624.2 ± 5.0 | 1710.5 ± 6.0 | 1.568 ± 0.03 | 56.3 ± 5.0 | 1.23 ± 0.1 | 81.03 ± 0.5 |
| Eudistomide B [29] | 770.05 | 205.82 ± 0.4 | 625.3 ± 5.0 | 1720.3 ± 6.0 | 1.572 ± 0.03 | 57.2 ± 5.0 | 1.23 ± 0.1 | 81.59 ± 0.5 |
| Mollamide [30] | 808.04 | 220.10 ± 0.5 | 613.0 ± 7.0 | 1634.0 ± 8.0 | 1.637 ± 0.05 | 50.4 ± 7.0 | 1.31 ± 0.1 | 87.25 ± 0.5 |
| Patellin 3 [31] | 943.25 | 254.84 ± 0.5 | 745.0 ± 7.0 | 1927.3 ± 8.0 | 1.599 ± 0.05 | 44.7 ± 7.0 | 1.26 ± 0.1 | 101.02 ± 0.5 |
| Patellin 4 [31] | 929.22 | 250.23 ± 0.5 | 729.0 ± 7.0 | 1888.7 ± 8.0 | 1.602 ± 0.05 | 45.0 ± 7.0 | 1.27 ± 0.1 | 99.19 ± 0.5 |
| Wewakazole [32] | 1141.29 | 298.59 ± 0.4 | 824.4 ± 5.0 | 2423.6 ± 6.0 | 1.644 ± 0.03 | 74.6 ± 5.0 | 1.38 ± 0.1 | 118.37 ± 0.5 |
| Wewakazole B [33] | 1127.26 | 293.98 ± 0.4 | 807.4 ± 5.0 | 2383.8 ± 6.0 | 1.648 ± 0.03 | 75.9 ± 5.0 | 1.39 ± 0.1 | 116.54 ± 0.5 |
| Wewakpeptin A [34] | 984.28 | 265.44 ± 0.4 | 836.9 ± 5.0 | 2247.5 ± 6.0 | 1.547 ± 0.03 | 51.9 ± 5.0 | 1.17 ± 0.1 | 105.23 ± 0.5 |
| Wewakpeptin B [34] | 988.31 | 267.63 ± 0.4 | 852.0 ± 5.0 | 2268.1 ± 6.0 | 1.540 ± 0.03 | 50.2 ± 5.0 | 1.15 ± 0.1 | 106.09 ± 0.5 |

Molar refractivity (MR^20^); molar volume (MV^20^); parachor (P_r_); index of refraction (n^20^); surface tension (γ^20^); density (d^20^); polarizability (α).

**References**

1. Fernandez, R.; Omar, S.; Feliz, M.; Quinoa, E.; Riguera, R. Malaysiatin, the first cyclic heptapeptide from a marine sponge. *Tetrahedron Lett.* **1992**, *33*, 6017–6020.
2. Brennan, M.R.; Costello, C.E.; Maleknia, S.D.; Pettit, G.R.; Erickson, K.L. Stylopeptide 2, a proline-rich cyclodecapeptide from the sponge *Stylotella* sp. *J. Nat. Prod.* **2008**, *71*, 453–436.
3. Afifi, A.H.; El-Desoky, A.H.; Kato, H.; Mangindaan, R.E.P.; de Voogd, N.J.; Ammar, N.M.; Hifnawy, M.S.; Tsukamoto, S. Carteritins A and B, cyclic heptapeptides from the marine sponge *Stylissa carteri*. *Tetrahedron Lett.* **2016**, *57*, 1285–1288.
4. Arai, M.; Yamano, Y.; Fujita, M.; Setiawan, A.; Kobayashi, M. Stylissamide X, a new proline-rich cyclic octapeptide as an inhibitor of cell migration, from an Indonesian marine sponge of *Stylissa* sp. *Bioorg. Med. Chem. Lett.* **2012**, *22*, 1818–1821.
5. [Zhan, K.X](http://www.ncbi.nlm.nih.gov/pubmed/?term=Zhan%20KX%5BAuthor%5D&cauthor=true&cauthor_uid=25490132).; [Jiao, W.H](http://www.ncbi.nlm.nih.gov/pubmed/?term=Jiao%20WH%5BAuthor%5D&cauthor=true&cauthor_uid=25490132).; [Yang, F](http://www.ncbi.nlm.nih.gov/pubmed/?term=Yang%20F%5BAuthor%5D&cauthor=true&cauthor_uid=25490132).; [Li, J](http://www.ncbi.nlm.nih.gov/pubmed/?term=Li%20J%5BAuthor%5D&cauthor=true&cauthor_uid=25490132).; [Wang, S.P](http://www.ncbi.nlm.nih.gov/pubmed/?term=Wang%20SP%5BAuthor%5D&cauthor=true&cauthor_uid=25490132).; [Li, Y.S](http://www.ncbi.nlm.nih.gov/pubmed/?term=Li%20YS%5BAuthor%5D&cauthor=true&cauthor_uid=25490132).; [Han, B.N](http://www.ncbi.nlm.nih.gov/pubmed/?term=Han%20BN%5BAuthor%5D&cauthor=true&cauthor_uid=25490132).; [Lin, H.W](http://www.ncbi.nlm.nih.gov/pubmed/?term=Lin%20HW%5BAuthor%5D&cauthor=true&cauthor_uid=25490132). Reniochalistatins A–E, cyclic peptides from the marine sponge *Reniochalina stalagmitis*. [*J. Nat. Prod.*](http://www.ncbi.nlm.nih.gov/pubmed/25490132) **2014**, *77*, 2678–2684.
6. Napolitano, A.; Bruno, I.; Riccio, R.; Gomez-Paloma, L. Synthesis, structure, and biological aspects of cyclopeptides related to marine phakellistatins 7–9. *Tetrahedron* **2005**, *61*, 6808–6815.
7. Pettit, G.R.; Cichacz, Z.; Barkoczy, J.; Dorsaz, A.C.; Herald, D.L.; Williams, M.D.; Doubek, D.L.; Schmidt, J.M.; Tackett, L.P.; Brune, D.C.; et al. Isolation and structure of the marine sponge cell growth inhibitory cyclic peptide phakellistatin 1. *J. Nat. Prod.* **1993**, *56*, 260–267.
8. Zhang, H.J.; Yi, Y.H.; Yang, G.J.; Hu, M.Y.; Cao, G.D.; Yang, F.; Lin, H.W. Proline-containing cyclopeptides from the marine sponge *Phakellia fusca*. *J. Nat. Prod.* **2010**, *73*, 650–655.
9. Cheng, Y.X.; Zhou, L.L.; Yan, Y.M.; Chen, K.X.; Hou, F.F. Diabetic nephropathy-related active cyclic peptides from the roots of *Brachystemma calycinum*. *Bioorg. Med. Chem. Lett.* **2011**, *21*, 7334–7439.
10. Kita, M.; Gise, B.; Kawamura, A.; Kigoshi, H. Stylissatin A, a cyclic peptide that inhibits nitric oxide production from the marine sponge *Stylissa massa*. *Tetrahedron Lett.* **2013**, *54*, 6826–6828.
11. Sun, J.; Cheng, W.; de Voogd, N.J.; Proksch, P.; Lin, W. Stylissatins B–D, cycloheptapeptides from the marine sponge *Stylissa massa*. *Tetrahedron Lett.* **2016**, in press.
12. Randazzo, A.; Piaz, F.D.; Orrù, S.; Debitus, C.; Roussakis, C.; Pucci, P.; Gomez-Paloma, L. Axinellins A and B: New proline-containing antiproliferative cyclopeptides from the Vanuatu sponge *Axinella carteri*. *Eur. J. Org. Chem.* **1998**, *11*, 2659–2665.
13. Tabudravu, J.N.; Morris, L.A.; Kettenes-van den Bosch, J.J.; Jaspars, M. Axinellin C, a proline-rich cyclic octapeptide isolated from the Fijian marine sponge *Stylotella aurantium*. *Tetrahedron* **2002**, *58*, 7863–7868.
14. Tabudravu, J.; Morris, L.A.; Kettenes-van den Bosch, J.J.; Jaspars, M. Wainunuamide, a histidine-containing proline-rich cyclic heptapeptide isolated from the Fijian marine sponge *Stylotella aurantium*. *Tetrahedron Lett.* **2001**, *42*, 9273–9276.
15. Pettit, G.R.; Clewlow, P.J.; Dufrense, C.; Doubek, D.L.; Cerny, R.L.; Rutzler, K. Antineoplastic agents. 193. Isolation and structure of the cyclic peptide hymenistatin 1. *Can. J. Chem.* **1990**, *68*, 708–711.
16. Vicente, J.; Vera, B.; Rodriguez, A.D.; Rodriguez-Escudero, I.; Raptis, R.G. Euryjanicin A: A new cycloheptapeptide from the Caribbean marine sponge *Prosuberites laughlini*. *Tetrahedron Lett.* **2009**, *50*, 4571–4574.
17. Vera, B.; Vicente, J.; Rodriguez, A.D. Isolation and structural elucidation of euryjanicins B–D, proline-containing cycloheptapeptides from the Caribbean marine sponge *Prosuberites laughlini*. *J. Nat. Prod.* **2009**, *72*, 1555–1562.
18. Williams, D.E.; Patrick, B.O.; [Behrisch, H.W](http://www.ncbi.nlm.nih.gov/pubmed/?term=Behrisch%20HW%5BAuthor%5D&cauthor=true&cauthor_uid=15787430).; [van soest, R](http://www.ncbi.nlm.nih.gov/pubmed/?term=Van%20Soest%20R%5BAuthor%5D&cauthor=true&cauthor_uid=15787430).; [Roberge, M](http://www.ncbi.nlm.nih.gov/pubmed/?term=Roberge%20M%5BAuthor%5D&cauthor=true&cauthor_uid=15787430).; [Andersen, R.J](http://www.ncbi.nlm.nih.gov/pubmed/?term=Andersen%20RJ%5BAuthor%5D&cauthor=true&cauthor_uid=15787430). Dominicin, a cyclic octapeptide, and laughine, a bromopyrrole alkaloid, isolated from the Caribbean marine sponge *Eurypon laughlini*. *J. Nat. Prod.* **2005**, *68*, 327–330.
19. Woo, J.K.; Jeon, J.E.; Kim, C.K.; Sim, C.J.; Oh, D.C.; Oh, K.B.; Shin, J. Gombamide A, a cyclic thiopeptide from the sponge *Clathria gombawuiensis*. *J. Nat. Prod.* **2013**, *76*, 1380–1383.
20. Kobayashi, J.; Tsuda, M.; Nakamura, T.; Mikami, Y.; Shigemori, H. Hymenamides A and B, new proline-rich cyclic heptapeptides from the okinawan marine sponge *hymeniacidon* sp. *Tetrahedron* **1993**, *49*, 2391–2402.
21. Tsuda, M.; Shigemori, H.; Mikami, Y.; Kobayashi, J. Hymenamides C–E, new cyclic heptapeptides with two proline residues from the Okinawan marine sponge *Hymeniacidon* sp. *Tetrahedron* **1993**, *49*, 6785–6796.
22. Kobayashi, J.; Nakamura, T.; Tsuda, M. Hymenamide F, new cyclic heptapeptide from marine sponge *Hymeniacidon* sp. *Tetrahedron* **1996**, *52*, 6355–6360.
23. Pettit, G.R.; Gao, F.; Cerny, R. Isolation and structure of axinastatin 4 from the western indian ocean marine sponge *Axinella* cf. carteri. *Heterocycles* **1993**, *35*, 711–718.
24. Pettit, G.R.; Gao, F.; Schmidt, J.M.; Cerny, R. Isolation and structure of axinastatin 5 from a Republic of Comoros marine sponge. *Bioorg. Med. Chem. Lett.* **1994**, *4*, 2935–2940.
25. Mohammed, R.; Peng, J.; Kelly, M.; Hamann, M.T. Cyclic heptapeptides from the jamaican sponge *Stylissa caribica*. *J. Nat. Prod.* **2006**, *69*, 1739–1744.
26. Rashid, M.A.; Gustafson, K.R.; Boswell, J.L.; Boyd, M.R. Haligramides A and B, two new cytotoxic hexapeptides from the marine sponge *Haliclona nigra*. *J. Nat. Prod.* **2000**, *63*, 956–959.
27. Sera, Y.; Adachi, K.; Fujii, K.; Shizuri, Y. A new antifouling hexapeptide from a palauan sponge, *Haliclona* sp. *J. Nat. Prod.* **2003**, *66*, 719–721.
28. Ibrahim, S.R.; Min, C.C.; [Teuscher, F](http://www.ncbi.nlm.nih.gov/pubmed/?term=Teuscher%20F%5BAuthor%5D&cauthor=true&cauthor_uid=20599387).; Ebel, R.; [Kakoschke, C](http://www.ncbi.nlm.nih.gov/pubmed/?term=Kakoschke%20C%5BAuthor%5D&cauthor=true&cauthor_uid=20599387).; [Lin, W](http://www.ncbi.nlm.nih.gov/pubmed/?term=Lin%20W%5BAuthor%5D&cauthor=true&cauthor_uid=20599387).; [Wray, V](http://www.ncbi.nlm.nih.gov/pubmed/?term=Wray%20V%5BAuthor%5D&cauthor=true&cauthor_uid=20599387).; [Edrada-Ebel, R](http://www.ncbi.nlm.nih.gov/pubmed/?term=Edrada-Ebel%20R%5BAuthor%5D&cauthor=true&cauthor_uid=20599387).; [Proksch, P](http://www.ncbi.nlm.nih.gov/pubmed/?term=Proksch%20P%5BAuthor%5D&cauthor=true&cauthor_uid=20599387). Callyaerins A–F and H, new cytotoxic cyclic peptides from the Indonesian marine sponge *Callyspongia aerizusa*. *Bioorg. Med. Chem.* **2010**, *18*, 4947–4956.
29. Whitson, E.L.; Ratnayake, A.S.; Bugni, T.S.; Harper, M.K.; Treland, C.M. Isolation, structure elucidation and synthesis of eudistomides A and B, lipopeptides from a fijian ascidian *Eudistoma* sp. *J. Org. Chem.* **2009**, *74*, 1156–1162.
30. Carroll, A.R.; Bowden, B.F.; Coll, J.C.; Hockless, D.C.R.; Skelton, B.W.; White, A.H. Studies of australian ascidians. IV. Mollamide, a cytotoxic cyclic heptapeptide from the compound ascidian *Didemnum molle*. *Aust. J. Chem.* **1994**, *47*, 61–69.
31. Carroll, A.R.; Coll, J.C.; Bourne, J.C.; MacLeod, J.K.; Zanriskie, T.M.; Ireland, C.M.; Bowden, B.F. Patellins 1-6 and Trunkamide A: Novel cyclic hexa-, hepta- and octa-peptides from colonial ascidians, *Lissoclinurn* sp. *Aust. J. Chem.* **1996**, *49*, 659–667.
32. Nogle, L.M.; Marquez, B.L.; Gerwick, W.H. Wewakazole, a novel cyclic dodecapeptide from a papua new guinea *Lyngbya majuscule*. *Org. Lett.* **2003**, *5*, 3–6.
33. Lopez, J.A.V.; Al-Lihaibi, S.S.; Alarif, W.M.; Abdel-Lateff, A.; Nogata, Y.; Washio, K.; Morikawa, M.; Okino, T. Wewakazole B, a cytotoxic cyanobactin from the cyanobacterium *Moorea producens* collected in the red sea. *J. Nat. Prod.* **2016**, *79*, 1213–1218.
34. Han, B.; Goeger, D.; Maier, C.S.; Gerwick, W.H. The Wewakpeptins, cyclic depsipeptides from a papua new guinea collection of the marine cyanobacterium *Lyngbya semiplena*. *J. Org. Chem.* **2005**, *70*, 3133–3139.
